# Proceedings of Boston Organon Society

**Published:** 1889-06

**Authors:** 


### PROCEEDINGS OF THE BOSTON ORGANON SOCIETY.

Meeting held March 28th.—Before beginning the reading of the *Organon,* Dr. Wesselhœft gave a very entertaining and instructive account of his recent trip among the Caribbean Islands. He spoke of their advantages in regard to climatic conditions, especially the island of Martinique. Upon this island almost any desirable altitude may be obtained.

Unfortunately, owing to the lack of intelligence or to negligence of ordinary precautions, small-pox is rife there, and the island is now and has been in a state of quarantine for two years. The climate of the other islands is very fine, Barbados in particular, but the island being rather more flat than mountainous different altitudes cannot be obtained. If steamship communication is ever established between Florida and these islands in order to avoid the dangers of Hatteras, this will be an excellent place to send our phthisical patients. He spoke of two cases of wonderful recovery of phthisis at Barbados, one of which was carried on a cot from the steamer to the hotel, and who now were seemingly as well as anybody. They had been there about two years.

The reading of the *Organon* was then commenced by Dr. Wesselhœft, beginning at the 148th Section.

Dr. Wesselhœft—This explanation never satisfied me, and, in fact, I do not care how it is done, but the most satisfactory explanation is that the remedy acts antidotally to the disease and not with it. The remedy meets the disease symptoms, if it is a simillimum it cures them, if it is not a simillimum but a simile, it partially meets them or meets them at an angle, and another set of symptoms, the resultant of these two forces, is generated, and a remedy must be selected for these, and so the patient is zig-zagged into health as Dr. Lippe used to say. These things, however, are all theoretical, and will never explain how it happens any more than we can explain how life happens.

Dr. Bell—The only value of a theoretical explanation is in regard to the dose, whether a larger or smaller dose is necessary.

Patients often ask for a powerful dose. The remedy calls into action the vital force as the pressure on an electric button sets in action the forces that ring a bell, or cause a large explosion.

Dr. Kennedy—We should separate the principle of selecting the remedy from the principle of its action. It always seemed to me that an imitation of the vital force causes disease. If the vital force is strong enough to overcome it alone the patient gets well without medicine. If it is not strong enough the drug helps it.

Foot-note, 101. Dr. Wesselhœft—What could be more scathing than this foot-note, and it is just as true now.

149. Dr. Wesselhœft—How quickly we relieve with the specific remedy in cases that last for weeks under other treatment! A follicular tonsillitis will recover in forty-eight hours with no after effects; it will never get well so quickly under mercurial treatment or swabbing with Corrosive Sublimate, etc. How often do we see in cases of recent wettings an inflammatory process cut short if we get there in time? I recall a case of sickness after eating pork, in which relief was obtained in two hours after taking Puls. The patient had always been sick days before in similar cases. Hahnemann makes a distinction between acute and long standing drug disease. There is great difficulty in finding the remedy in cases of drug complication. With patients that have been treated allopathically for syphilis, it is always a long story of wrongly applied remedies, and not the result of the disease. We have seen many cases come with the worst kind of primary symptoms that have been cured with no secondary symptoms.

Cases are often made incurable by a wrongly selected remedy, and a remedy partially homœopathic may do just as much harm as an antipathic remedy. The disease is nothing compared to the ordinary method of cure.

Dr. Bell—I saw a case this morning that has been under treatment since last August. He came at that time with a primary hunterian chancre that he had then had for two months. He had been treated carefully out West before coming to us, had been given one dose of Mercurius$^{200}$ and had been told to let the thing alone.

I examined him carefully to-day; he is perfectly free from disease; his glands are all free. He received about three doses of medicine and never had any secondary symptoms, but a few maculæ.

Dr. Wesselhœft—Now, when they are giving Corrosive Sublimate and large doses of Iodide of Potassium, it takes three or four years to get a patient rid of such a drug-complicated disease. That is what Hahnemann meant by a drug disease, and it takes three or four times as long to effect a cure after a drug poisoning. We have cases of fever and ague that after being cured homœopathically can return to malarial districts without contracting the disease again.

Now, old-school physicians have always said when we reported such cases that our diagnosis was wrong, they do not say so now; they know that we can make as good a diagnosis as they can. Some time ago I had a case of gonorrhœa that had been treated by injections, the urethra stretched for stricture, and as a last resort the urethra was to be cut; then the patient came to me. The injection had caused a prostatitis, and, in addition, there were several condylomata present. After a dose of Pulsatilla the difficulty of urination passed away, together with the prostatitis and the condylomata, and the discharge was re-established, proving effectually to the patient what had been the cause of all his troubles.

He told his former physician, in a few weeks, how much better he was, and how all the distressing symptoms indicative of stricture had disappeared, and all that worthy could say, was, "Well, he must have used some very powerful medicine!"

Dr. Bell—We would not have cases of tertiary syphilis if the patients were properly treated in the first place.

Dr. Wesselhœft—I recall a case of syphilis complicated with malaria, with profuse hemorrhages from the kidneys; he was very anæmic but is now perfectly well.

150. Dr. Wesselhœft—Hahnemann means that we are not to give a remedy for every little thing. Adjourned to April 11th.

